# The utility of electron microscopy in detecting asbestos fibers and particles in BALF in diffuse lung diseases

**DOI:** 10.1186/s12890-017-0415-5

**Published:** 2017-04-21

**Authors:** Takashi Kido, Yasuo Morimoto, Kazuhiro Yatera, Hiroshi Ishimoto, Takaaki Ogoshi, Keishi Oda, Kei Yamasaki, Toshinori Kawanami, Shohei Shimajiri, Hiroshi Mukae

**Affiliations:** 1 Department of Respiratory Medicine, University of Occupational and Environmental Health, 1-1 Iseigaoka, Yahatanishi-ku, Kitakyushu, Fukuoka Japan; 2 Department of Occupational Pneumology, University of Occupational and Environmental Health, 1-1 Iseigaoka, Yahatanishi-ku, Kitakyushu, Fukuoka Japan; 3 Second Department of Internal Medicine, Nagasaki University School of Medicine, 1-7-1 Sakamoto, Nagasaki, Japan

**Keywords:** Asbestos, Bronchoalveolar lavage, Diffuse lung diseases, Electron microscopy, Elements, Light microscopy, Occupational exposure

## Abstract

**Background:**

In patients with diffuse lung diseases, differentiating occupational lung diseases from other diseases is clinically important. However, the value of assessing asbestos and particles in bronchoalveolar lavage fluid (BALF) in diffuse lung diseases by electron microscopy (EM) remains unclear. We evaluated the utility of EM in detecting asbestos fibers and particles in patients with diffuse lung diseases.

**Methods:**

The BALF specimens of 107 patients with diffuse lung diseases were evaluated. First, detection of asbestos by EM and light microscopy (LM) were compared. Second, the detection of asbestos using surgically obtained lung tissues of 8 of 107 patients were compared with the results of EM and LM in BALF. Third, we compared the results of mineralogical components of particles in patients with (*n =* 48) and without (*n =* 59) a history of occupational exposure to inorganic dust.

**Results:**

BALF asbestos were detected in 11 of 48 patients with a history of occupational exposure by EM; whereas asbestos as asbestos bodies (ABs) were detected in BALF in 4 of these 11 patients by LM. Eight of 107 patients in whom lung tissue samples were surgically obtained, EM detected BALF asbestos at a level of >1,000 fibers/ml in all three patients who had ABs in lung tissue samples by LM at a level of >1,000 fibers/g. The BALF asbestos concentration by EM and in lung tissue by LM were positively correlated. The particle fractions of iron and phosphorus were increased in patients with a history of occupational exposure and both correlated with a history of occupational exposure by a multiple regression analysis.

**Conclusions:**

EM using BALF seemed to be superior to LM using BALF and displayed a similar sensitivity to LM using surgically-obtained lung tissue samples in the detection of asbestos. Our results also suggest that detection of elements, such as iron and phosphorus in particles, is useful for evaluating occupational exposure. We conclude that the detection of asbestos and iron and phosphorus in particles in BALF by EM is very useful for the evaluation of occupational exposure.

## Background

Differentiating occupational lung diseases including asbestosis from other diffuse lung diseases, such as idiopathic pulmonary fibrosis (IPF), is important in patients with diffuse lung diseases, not only for diagnostic and treatment but also for legal, ecological and social reasons [1].

The detection of asbestos bodies (ABs) in bronchoalveolar lavage fluid (BALF) by light microscopy (LM) is useful for diagnosing asbestos-related lung diseases, but is sometimes undetectable, even in patients with heavy asbestos exposure [2–7]. Surgical biopsies are also considered useful for evaluating asbestos exposure and diagnosing asbestosis; however, it is a relatively invasive procedure and can be difficult to perform, especially in patients with an impaired pulmonary function [8].

Electron microscopy (EM) can quantify mineral fibers and particles in the sample and also detect detailed elements via X-ray analytical EM [8]. However, the Helsinki Criteria and the criteria of the American Thoracic Society (ATS) and European Respiratory Society (ERS) include no diagnostic criteria pertaining to the use of EM in the detection of asbestos fibers (uncoated asbestos fibers and ABs; AFs) in BALF [8–10]. Furthermore, the utility of detecting mineralogical elements in BALF by EM in diagnosing diffuse lung diseases is unclear so far. Therefore, we investigated the diagnostic utility of detecting AFs and the mineralogical elements in BALF by EM in patients with diffuse lung diseases.

## Methods

### Patients

Between January 2012 to December 2014, 107 patients who underwent bronchoalveolar lavage for the diagnosis of diffuse lung diseases were enrolled. This study was approved by the Human and Animal Ethics Review Committee of the University of Occupational and Environmental Health, Japan (Approval number: H23-120). Written informed consent was obtained from all patients.

### Clinical characteristics assessment

The patients’ clinical characteristics, including their occupational exposure histories were recorded. The official statements of the ATS/ERS/Japanese Respiratory Society/Latin American Thoracic Association of IPF and the ATS/ERS classification of IIPs were used for the definition of idiopathic interstitial pneumonias (IIPs) [11, 12]. For the diagnosis of asbestosis, we used the criteria outlined in the report delivered by the asbestosis committee of the American Pathologists and Pulmonary Pathology Society [13].

### Bronchoalveolar lavage (BAL)

BAL was performed using flexible bronchofiberscopy after local anesthesia with lidocaine. Three 50 ml fractions of sterile saline were instilled into the right middle lobe or the left lingular segment of the lung. BALF was retrieved by gentle syringe suction and put into sterile containers.

### Preparation for fiber and particle detection

Technicians and observers were blinded to clinical information when evaluating fibers and particles. BALF (10 ml) was filtered by a membrane filter (Nuclepore Track-Etch Membrane, Whatman Schleicher & Schuell, UK), then two times of low temperature ashing were performed over one day in an oxygen plasma asher (LTA-102, Yanaco, Japan). A quarter of each filter sample was used for an EM analysis using a scanning electron microscope (SEM) and a transmission electron microscope (TEM); the remaining half was used for a LM analysis.

Lung tissue specimens were obtained by surgical biopsy or autopsy. Samples were taken from the peripheral part of the lung, not including the tumor when neoplastic lesions were sampled. Autopsy lung samples were collected from all of the lobes; the average value was calculated to determine the concentration of ABs. Formalin-fixed lung tissue was lyophilized overnight, ashed three times, and then subjected to a LM analysis.

### The detection of ABs by LM

Half of each sample on the filter was cut, ashed overnight at low temperature, and resuspended in 3 ml of distilled water. Each sample was aspirated through a membrane filter (mixed cellulose ester; diameter: 25 mm; pore size: 0.45 μm) (Advantec Toyo Roshi Kaisha Ltd. Japan) and dried. The filter was attached to a glass slide using acetone vapor with the dust side facing the glass slide, as described previously [14]. ABs were then counted under LM (magnification: ×400). Only typical ABs, such as fibers of 2 to 5 μm in width and 20 to 50 μm in length with a rod or dumbbell shape and multiple segmentation, were counted by trained technicians. The concentration of ABs/ml or ABs/g (g of dry weight lung) was calculated, as described previously [3, 5, 15, 16].

### Counting fibers and particles by SEM

A quarter of each sample on the filter was cut, and fixed on an aluminum stand with double adhesive carbon tape. Osmium evaporation was applied to the surface of the sample on the carbon tape. Fibers and particles were then counted under a SEM (S-4500 Hitachi, Japan) and the concentration was calculated [14].

The analysis was performed at × 3,000 magnification. A particle with a length-to-width ratio of >3 was considered to be a fiber; the numbers of fibers were counted in at least 100 randomly selected fields. If the total number of fibers was <20, then all of the fibers in all of the fields were counted. Non-fibrous particles were counted until 100 particles were reached in the selected fields (minimum: 3 fields).

### AFs and particles identification by TEM

**Fig. 1:**
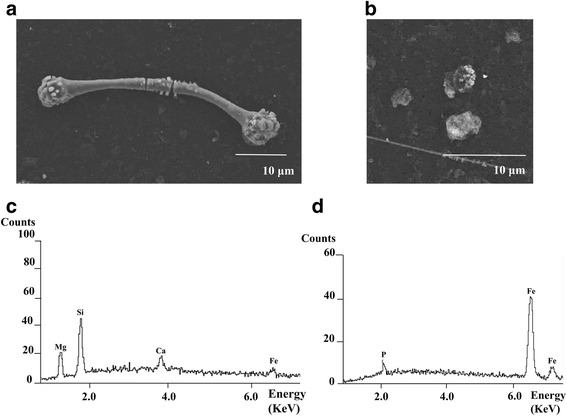
An example case of the analysis by electron microscopy. The fibers and particles were divided into ferruginous bodies (**a**), uncoated fibers, and particles (**b**) based on the morphological findings. A mineralogical analysis revealed that the ferruginous body contained magnesium (Mg) and silicon (Si), calcium (Ca) and iron (Fe), suggesting it to be an actinolite asbestos body (**c**). Particles containing Fe and phosphorus (P) (**d**)

A quarter of each sample on the filter was cut, and fixed on an aluminum stand with double adhesive carbon tape. Carbon evaporation was applied to the surface of the sample on the carbon tape. AFs and particles were identified using a TEM (JEM-2000 EX, JOEL, Japan), and the mineralogical elements were detected by an energy dispersive X-ray spectrometry analysis (Oxford Isis 300, UK) (Fig. 1) [14, 17, 18].

### Calculation of the concentration of AFs and particles containing each element

The concentration of AFs and particles and relative fraction of particles containing each element (e.g. if 60 particles contained iron and silicon, and 140 particles contained silicon in 200 particles, then the fraction of particles containing iron was 30.0% and the fraction of particles containing silicon was 100%) were calculated according to the SEM and TEM data (Fig. 1).

Patients were divided into two groups based on their history of occupational exposure to inorganic dust (the exposure-positive and exposure-negative groups). The exposure-positive group was additionally divided into two subgroups based on the detection of AFs by EM in their BALF samples (the AF-positive and AF-negative subgroups).

### Statistical analysis

The categorical data were analyzed using the *χ* ^2^ test or Fisher’s exact test, as appropriate. The Mann–Whitney *U* test was used to compare continuous variables of the exposure-positive and exposure-negative groups. The Kruskal-Wallis test was used to compare the continuous variables in three groups; significant values were compared among the groups by Dunn’s test. Spearman’s rank correlation coefficients (*rS*) were calculated to identify associations between the concentrations of ABs in the surgically-obtained lung tissue samples and the detection of asbestos in BALF (AFs and ABs detected by EM, and ABs detected by LM). A multiple regression analysis was performed to examine the fractions of particles that included iron and phosphorus. Values of *p* < 0.05 were considered to be statistically significant in all tests. The IBM SPSS 22.0 (IBM SPSS, Armonk, New York, USA) and Stat Flex 6.0 (Artech, Osaka, Japan) software programs were used for the statistical analyses.

## Results

A total of 107 patients with diffuse lung diseases were enrolled. Table 1 shows the baseline characteristics of these patients. Sixty five patients were male, 59 patients had a smoking history, and 48 patients had a history of occupational exposure to inorganic dust, including a history of working in building construction, electrical construction, foundry molding, mining, a ship’s hold, asbestos factory, automobile factory, cement factory, train factory, piping construction and water supply construction.

**Table 1:** The baseline characteristics

	*n =* 107
Male	65 (60.7)
Age, years	63.4 (1.1)
Positive for smoking history	59 (55.1)
(Current smoker)	18 (16.8)
(Ex-smoker)	41 (38.3)
Brinkman Index	530.9 (79.0)
History of occupational exposure positive	48 (44.9)
Lung function test	
VC, ml	2,544 (103.5)
VC,% predicted	80.2 (2.5)
FEV_1_, ml	2,012.2 (78.5)
FEV_1_%	77.8 (1.5)
D_LCO,_% predicted	69.0 (3.0)
KL-6 in serum, U/ml	1,173.7 (111.9)
CT findings	
Ground glass opacity or/and reticular shadow	72 (67.2)
Consolidation	31 (29.0)
Bronchiectasis	42 (39.2)
Honeycomb	24 (22.4)
Emphysema	29 (27.1)
Plaque	7 (6.5)
BALF	
Total cell, ×10^5^/ml	6.6 (2.2)
Macrophage,%	68.4 (2.2)
Lymphocyte,%	15.4 (1.7)
Eosinophil,%	5.3 (0.9)
Neutrophil,%	11.5 (1.9)
CD4/8	2.7 (0.3)
Diagnosis of diffuse lung diseases	
Asbestosis	6 (5.6)
IPF	26 (24.2)
IIPs other than IPF	14 (13.1)
CHP	10 (9.3)
CEP	7 (6.5)
CTD with diffuse lung diseases	14 (13.1)
Sarcoidosis	15 (14.0)
Other diffuse lung diseases	15 (14.0)

Data are presented as n (%) or mean (SE), unless otherwise stated

*BALF* bronchoalveolar lavage fluid, *CEP* chronic eosinophilic pneumonia, *CHP* chronic hypersensitivity pneumonia, *CT* computed tomography, *CTD* connective tissue diseases, *D*_*LCO*_ diffusing capacity of the lung of carbon monoxide, *Exposure-positive* positive history of occupational exposure, *Exposure-negative* negative history of occupational exposure, *IIPs* Idiopathic interstitial pneumonias, *IPF* idiopathic pulmonary fibrosis, *KL-6* Krebs von den Lungen-6

**Fig. 2:**
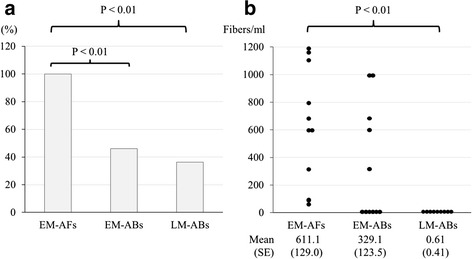
The detection rate and concentration of AFs and ABs in the 11 of 107 patients who were detected AFs by EM. ABs were only detected in 5 of these 11 patients (45.5%) by EM, and in 4 of these 11 patients (36.3%) by LM (*P* < 0.01) (**a**). The actual number of concentrations of AFs and ABs by EM and LM in BALF, suggest that EM has an approximately 1,000-fold-greater ability to detect AFs and a 500-fold-greater ability to detect ABs than LM does for ABs in BALF (**b**). Abbreviations: ABs, Asbestos bodies; AFs, uncoated asbestos fibers and ABs; BALF, BAL fluid; EM, electron microscopy; LM, light microscopy

The analysis of BALF by EM detected AFs in 11 patients (22.9%) of the exposure-positive group, including patients with asbestosis, IPF and chronic hypersensitivity pneumonia (Table 2). Eleven patients in whom AFs were detected had a history of occupational exposure to inorganic dust through building construction (*n =* 6), foundry molding (*n =* 2), electrical construction (*n =* 1), asbestos factory (*n =* 1), train factory (*n =* 1) and water supply construction (*n =* 1). In the 11 patients who were detected AFs by EM, ABs were only detected in 5 and 4 of 11 patients (45.5% and 36.3%) by EM and LM, respectively (Fig. 2a). Other than these 11 patients, ABs were not detected by LM. Figure 2b shows the actual concentration of AFs and ABs by EM and LM in BALF, suggesting that EM has an approximately 1,000-fold-greater ability to detect AFs and a 500-fold-greater ability to detect ABs than LM does for ABs in BALF.

**Table 2:** The diagnosis of lung diseases in cases where asbestos fibers were detected in bronchoalveolar lavage fluid by electron microscopy in the exposure-positive and exposure-negative groups

Diagnosis	Exposure-positive	Exposure-negative	*P*
Total	11/48 (22.9)	0/59 (0)	<0.001
Asbestosis	6/6 (100)	0/0 (0)	
IIPs	4/22 (18.2)	0/18 (0)	
(IPF)	{4/16 (25.0)}	{0/10 (0)}	
CHP	1/7 (14.2)	0/3(0)	
CEP	0/0 (0)	0/7(0)	
CTD	0/2 (0)	0/12(0)	
Sarcoidosis	0/7(0)	0/8(0)	
Other diffuse ILD	0/4(0)	0/11(0)	

Data are presented as number of patients detected asbestos fibers/total number of patients (%)

*AFs* uncoated asbestos fibers and asbestos bodies, *CHP* chronic hypersensitivity pneumonia, *Exposure-positive* positive history of occupational exposure to inorganic dust, *Exposure-negative* negative history of occupational exposure to inorganic dust, *IPF* idiopathic pulmonary fibrosis

Table 3 shows the concentrations of ABs determined by LM in the lung tissue samples of eight patients who underwent a surgical biopsy. ABs were detected at a level of >1,000 ABs/g in lung tissue samples by LM in three patients, and AFs were also detected at a level of >1,000 AFs/ml in BALF by EM in all of these three patients. There was a significant association between the concentration of ABs detected in lung tissues by LM and the BALF concentration of AFs detected by EM (*rS* = 0.913, *p* < 0.01) and BALF ABs detected by LM (*rS* = 0.791, *p* < 0.05), while BALF concentration of ABs detected by LM was not correlated with the concentration of ABs detected in lung tissue specimens by LM (*rS* = 0.514) (Table 4).

**Table 3:** Comparison of detection of asbestos fibers in surgically-obtained lung tissues and in bronchoalveolar lavage fluid by electron and light microscopy

		Lung tissue	BALF	
		Light Microscopy	Electron Microscopy	Light Microscopy
Diagnosis	Objective for lung biopsy	ABs /g (Lobe)	AFs (ABs) /ml	ABs /ml
Asbestosis	Lung cancer	14,004.50 (RL)	1,170 (320)	0
Asbestosis	Lung cancer	4,802.40 (RU)	1,190 (990)	0.84
IPF	Lung cancer	2,062 (LL)	1,100 (980)	0.4
IPF	Lung cancer	166 (LU)	130 (0)	0
IPF	Lung cancer	65.6 (RL)	0 (0)	0
IPF	Lung cancer	0 (RL)	0 (0)	0
IPF	Diagnosis	90.4 (LU)	0 (0)	0
CHP	Autopsy	64 (RL)	0 (0)	0

*ABs* Asbestos bodies, *AFs* uncoated asbestos fibers and ABs, *BALF* bronchoalveolar lavage fluid, *CHP* chronic hypersensitivity pneumonia, IPF idiopathic pulmonary fibrosis, *LL* left lower, *LU* left upper, *RL* right lower, *RU* right upper

**Table 4:** Spearman’s rank correlation coefficient between the level of asbestos fibers in the lung tissue samples and in bronchoalveolar lavage fluid by electron and light microscopy

Association with the concentration of ABs detected in surgically-obtained lung tissues obtained by light microscopy	*rS*	*P*
Concentration of AFs detected in BALF by electron microscopy	0.913	<0.01
Concentration of ABs detected in BALF by electron microscopy	0.791	<0.05
Concentration of ABs detected in BALF by light microscopy	0.514	NS

*ABs* Asbestos bodies, *AFs* uncoated asbestos fibers and ABs, *BALF* bronchoalveolar lavage fluid, *CHP* chronic hypersensitivity pneumonia, *IPF* idiopathic pulmonary fibrosis, *NS* not significant, *rS* Spearman’s rank correlation coefficient

Table 5 shows the results of particle analyses in BALF by EM in two subgroups (the AF-positive and AF-negative subgroups) in the exposure-positive and exposure-negative groups. The fractions of iron and phosphorus were significantly higher in both the AFs-positive and AFs-negative subgroups than in the exposure-negative group. The fraction of calcium was significantly lower in the AFs-negative subgroup than in the exposure-negative group. A multiple regression analysis of the fractions of particles containing iron and phosphorus revealed that an occupational exposure history and age were correlated with the fraction of particles containing iron (Table 6), and the fraction of particles containing phosphorus was correlated with a history of occupational exposure (Table 7).

**Table 5:** The comparison of the results of analysis of particles in bronchoalveolar lavage fluid by electron microscopy

	Exposure-positive (*n =* 48)		Exposure-negative (*n =* 59)
AFs in electron microscope	AFs-positive (*n =*11)	AFs-negative (*n =* 37)	AFs-negative (*n =* 59)
Detection rate of particles	11 (100)	37 (100)	59 (100)
Concentration of particles, ×10^6^/ml	2.75 ± 2.27	2.43 ± 1.88	3.27 ± 3.94
Silicon,%	88.0 ± 12.3	92.2 ± 12.7	94.2 ± 10.4
Iron,%	16.2 ± 18.3^*^	9.0 ± 16.8^**^	1.0 ± 2.8
Phosphorus,%	15.5 ± 14.6^*^	11.4 ± 17.7^**^	3.3 ± 9.1
Aluminum,%	5.3 ± 6.7	3.2 ± 3.7	1.8 ± 2.5
Sodium,%	0.8 ± 1.4	1.5 ± 3.9	2.9 ± 7.1
Titanium,%	0.3 ± 1.1	0.6 ± 1.4	0.7 ± 3.5
Magnesium,%	0.2 ± 0.6	0.8 ± 2.6	0.6 ± 1.6
Zinc,%	0.0 ± 0.0	0.0 ± 0.2	0.7 ± 1.8
Calcium,%	0.0 ± 0.0	0.1 ± 0.5^**^	2.2 ± 9.3
Sulfur,%	0.0 ± 0.0	0.2 ± 0.9	0.0 ± 0.2
Copper,%	0.0 ± 0.0	0.0 ± 0.3	0.0 ± 0.0
Potassium,%	0.0 ± 0.0	0.3 ± 0.9	0.6 ± 2.0
Chlorine,%	0.0 ± 0.0	0.0 ± 0.0	0.0 ± 0.2
Sulfur,%	0.0 ± 0.0	0.0 ± 0.0	10.0 ± 31.6

Data are presented as n (%) or mean ± SD, unless otherwise stated

^*^ *P* < 0.01 versus AFs-negative subgroup; ^**^ *P* < 0.05 versus exposure-negative group

*AFs* uncoated asbestos fibers and asbestos bodies, *Exposure-positive* positive history of occupational exposure to inorganic dust, *Exposure-negative* negative history of occupational exposure to inorganic dust

**Table 6:** The univariate and multiple regression analysis for the fraction of iron of particles in bronchoalveolar lavage fluid

	Univariate regression analysis		Multiple regression analysis	
Variables	Standardized beta Coefficient	*P*	Standardized beta Coefficient	*P*
History of occupational exposure positive	0.288	0.011	0.337	<0.001
Positive detection of AFs in BALF by electron microscope	0.17	0.089	0.184	0.055
Age, (yr)	−0.185	0.049	−0.181	0.045
Male	0.071	0.537		
Smoking history positive	0.04	0.719		

*AFs* uncoated asbestos fibers and asbestos bodies, *BALF* bronchoalveolar lavage fluid

**Table 7:** The univariate and multiple regression analysis for the fraction of phosphorus of particles in bronchoalveolar lavage fluid

	Univariate regression analysis		Multiple regression analysis	
Variables	Standardized beta Coefficient	*P*	Standardized beta Coefficient	*P*
History of occupational exposure to inorganic dust	0.276	0.019	0.325	0.001
Positive detection of AFs in BALF by electron microscope	0.081	0.437		
Age, (yr)	−0.127	0.196		
Male	0.016	0.892		
Smoking history positive	0.066	0.572		

*AFs* uncoated asbestos fibers and asbestos bodies, *BALF* bronchoalveolar lavage fluid

## Discussion

BALF AFs was observed in 11 of 107 patients using the EM, and BALF ABs was detected in 4/11 patients (36.3%) by LM. In addition, a significant correlation of AFs and ABs concentration in BALF by EM and ABs in lung tissues by LM was noted. EM detected BALF AFs at a level of >1,000 fibers/ml in all three patients who had ABs in lung tissue samples by LM at a level of >1,000 fibers/g. These results suggest that using BALF, EM was superior to LM to detect AFs, and the sensitivity of EM in detecting AFs in BALF was similar to that of LM using surgically-obtained lung tissue samples. In addition, the elemental fractions of particles containing iron and phosphorus were significantly higher in the exposure-positive group than in the exposure-negative group, and a multiple regression analysis revealed that iron and phosphorus fractions were significantly correlated with an occupational exposure history. These results suggest that analysis including counting AFs and particles and evaluating their elements of BALF by EM is very useful for evaluating occupational exposure.

The Helsinki Criteria have a guideline for identifying a high probability of occupational asbestos exposure (>1 ABs/ml of BALF using LM) [10]. The ATS and the ERS also have similar guidelines [8, 9], However, these guidelines include no criteria for the analysis of BALF by EM. Obtaining BALF is much less invasive than surgical procedures [8, 19, 20], and EM can count small and thin fibers and particles with much higher resolution than LM and can also identify fiber types and elements with X-ray analysis [8]. Thus, the detection of AFs in BALF using EM appears to be a good diagnostic tool and it should be used when available. Indeed, LM only detected ABs in 4/11 BALF in which AFs were detected by EM in the present study (Fig. 2a). The most important reason for superior detecting ability of EM is that LM can detect only ABs from their morphology, while EM can also detect uncoated asbestos fibers by analyzing the elements [8, 9, 14, 16]. It is also said that many of the uncoated asbestos fibers detected by EM are too small to form Abs [4, 15, 16].

Quantitative lung tissue analysis is the gold standard for quantitating lung asbestos concentrations; a level of >1,000 ABs/g in a dry lung tissue sample is usually considered to be an indicator of nontrivial (usually occupational) exposure when determined by LM [8, 10]. The correlation between BALF AFs detected by EM and AFs in lung tissues has only been investigated in one report, which showed a good correlation between the concentration of BALF AFs detected and the AFs detected in lung tissues by EM [6]. A novel finding of the present study is that the correlation between the concentrations of BALF AFs detected by EM and ABs detected in lung tissues by LM (Table 4). To our knowledge, this is the first study to show the relationship between the concentrations of BALF AFs detected by EM and ABs detected in lung tissues by LM.

Lung tissue analysis by LM identified 3/11 patients with >1,000 ABs/g; the analysis of their BALF by EM showed >1,000 AFs/ml. These results were significantly correlated (*rS* = 0.913), suggesting the detection of 1,000 AFs/ml of BALF by EM may be indicative of occupational asbestos exposure. This is in line with the previous findings, which indicated the mean AFs/ml in BALF was 793 in asbestos-exposed subjects and that the lower confidence interval of occupational exposed workers was 1,054 fibers/ml [4, 21].

The clinical impact of the mineral components of particles in the diagnosis of diffuse lung diseases is unclear. This may be because such analysis requires EM and associated analytical techniques [22]. Our results show that the fraction of particles containing iron and phosphorus was significantly correlated with a history of occupational exposure. A few reports have shown the relationship between the mineral components of particles in BALF and occupational exposure to mineral dusts. Parion et al. showed significantly increased levels of iron in the BALF of 205 occupationally-exposed patients in comparison to 41 patients without occupational exposure, and Bernstein et al. also showed that iron was significantly increased in the BALF of 46 dental technicians with lung diseases in comparison to 41 white-collar workers with lung diseases [22, 23]. Our results showing an increase of elemental iron detected by EM in BALF in the exposure-positive group are consistent with these previous studies, and the detection of iron may be useful in screening for occupation-related lung diseases. It has also been shown that iron and phosphorus were detected in the same alveolar macrophages, and the detection of phosphorus may be useful for the evaluation of iron overload in cells [24, 25]. Because iron concentrations are known to increase under various conditions, it may be better to conduct an investigation with more variables and in a larger number of patients [26–29].

The present study is associated with several limitations. First, the lung diseases of the patients were heterogeneous; it would be preferable if the lung diseases of each group were relatively-uniform. Second, the analysis of lung tissue samples was only performed in eight patients; a larger number of patients would be preferred. Third, the analysis of BALF was only performed at our institution. Our results should therefore be confirmed in a multicenter trial [8].

## Conclusions

Our results suggest that EM using BALF was superior to LM using BALF, and the sensitivity of EM in detecting AFs in BALF was similar to that of LM using surgically-obtained lung tissue samples. Because surgical biopsy is more invasive, it is reasonable to consider the detection of AFs in BALF by EM to evaluate the occupational exposure. Another important finding is that detection of the elements of particles such as iron and phosphorus may be useful for assessing occupational exposure.

## Abbreviations

ABs: Asbestos bodies
AFs: uncoated asbestos fibers and ABs
ATS: American Thoracic Society
BAL: bronchoalveolar lavage
BALF: BAL fluid
CHP: Chronic hypersensitivity pneumonia
EM: Electron microscopy
ERS: European respiratory society
IIPs: Idiopathic interstitial pneumonias
IPF: Idiopathic pulmonary fibrosis
LM: Light microscopy
rS: Spearman’s rank correlation coefficient
SEM: Scanning electron microscope
TEM: Transmission electron microscope
